# The Effect of Baicalin as A PPAR Activator on Erythroid Differentiation of CD133^+^Hematopoietic Stem Cells in Umbilical Cord Blood

**DOI:** 10.22074/cellj.2015.508

**Published:** 2015-04-08

**Authors:** Parvaneh Abbasi, Karim Shamsasenjan, Ali Akbar Movassaghpour Akbari, Parvin Akbarzadehlaleh, Nima Dehdilani, Mostafa Ejtehadifar

**Affiliations:** 1 Hematology and Oncology Research Center, Tabriz University of Medical Sciences, Tabriz, Iran; 2 Blood Transfusion Research Center, High Institute for Research and Education in Transfusion Medicine, Tehran, Iran; 3 Department of Hematology, Faculty of Medical Sciences, Tabriz University of Medical Sciences, Tabriz, Iran; 4 Department of Pharmaceutical Biotechnology, Faculty of Pharmacy, Tabriz University of Medical Sciences, Tabriz, Iran

**Keywords:** PPARγ, Baicalin, Hematopoietic Stem Cell, Erythropoiesis

## Abstract

**Objective:**

The peroxisome proliferator-activated receptors (PPARs) are a group of nu- clear receptor proteins whose functions as transcription factors regulate gene expres- sions. PPARs play essential roles in the regulation of cellular differentiation, development, and metabolism (carbohydrate, lipid, protein), and tumorigenesis of higher organisms. This study attempts to determine the effect of baicalin, a PPARγ activator, on erythroid differentiation of cluster of differentiation 133^+^(CD133^+^) cord blood hematopoietic stem cells (HSCs).

**Materials and Methods:**

In this experimental study, in order to investigate the effects of the PPARγ agonists baicalin and troglitazone on erythropoiesis, we isolated CD133^+^ cells from human umbilical cord blood using the MACS method. Isolated cells were cultured in erythroid-inducing medium with or without various amounts of the two PPARγ activa- tors (baicalin and troglitazone). Erythroid differentiation of CD133^+^cord blood HSCs were assessed using microscopic morphology analysis, flow cytometric analysis of erythroid surface markers transferrin receptor (TfR) and glycophorin A (GPA) and bycolony forming assay.

**Results:**

Microscopic and flow cytometric analysis revealed the erythroid differentiation of CD133^+^cord blood HSCs under applied erythroid inducing conditions. Our flow cytometric data showed that the TfR and GPA positive cell population diminished significantly in the presence of either troglitazone or baicalin. The suppression of erythroid differentiation in response to PPARγ agonists was dose-dependent. Erythroid colony-forming ability of HSC decreased significantly after treatment with both PPARγ agonists but troglitazone had a markedly greater effect.

**Conclusion:**

Our results have demonstrated that PPARγ agonists modulate erythroid dif- ferentiation of CD133^+^HSCs, and therefore play an important role in regulation of normal erythropoiesis under physiologic conditions. Thus, considering the availability and applica- tion of this herbal remedy for treatment of a wide range of diseases, the inhibitory effect of baicalin on erythropoiesis should be noted.

## Introduction

*Scutellaria baicalensis georgi* (English: Baikal skullcap or skullcap, Chinese: Huang qin, Japanese: Wong or Ogan) is a perennial herb from the Lamiaceae family cultivated in Japan, China and Korea. It is acclimated to grow in Germany and Poland. The dried root of this plant (*Radix Scutellariae*) contains high amounts of flavonoids that include14% of [5, 6 dihydroxy 8-methoxyflavone (baicalin), >7% of (5, 6 and 7 trihydroxy flavon (baicalein] and 7% of [5 and 7 dehydroxy 8-methoxyflavone (wogonin)], etc (1). Baicalinis the main flavonoid found in *Radix Scutellaria*. According to studies, this herb has multiple biological properties that include anticancer and protective effects against a variety of tissue and organ damages. To date, several studies have been conducted on the effects of baicalin on neural and musculoskeletal systems (2), its anti-inflammatory and antioxidant effects (3, 4), treatment of cardiac diseases (5), antimicrobial, antiviral and anticancer properties (6, 7) and its effect on cell cycle arrest in the G1 phase and apoptosis of cancer cells (8). In addition, its anti-proliferative effects in lung cancer (9), hepatocarcinoma (10), prostate cancer (11) and various cancer cell lines have been reported. The addition of baicalin to the K562 cell line inhibits erythroid differentiation and reduces colony-forming ability by affecting the Notch signaling pathway (12).

Baicalin is one of the peroxisome proliferator–activated receptor γ (PPARγ) agonists. PPARγ is a ligand–activated transcription factor that belonging to the nuclear receptor superfamily (13). The human nuclear receptor superfamily consists of 49 members found inside the nucleus or cytoplasm. This family of proteins is responsible for responding to thyroid or steroid hormones and plays a role in controlling development, homeostasis and organism metabolism (14). Nuclear receptors of PPARγ are expressed at high levels in adipose tissue and play a main role in adipocyte differentiation (15). In addition, expression of PPARγ nuclear receptors has been found in many cancer cell lines such as the fibroblast cell line, hepatocyte cell lines and epithelial cell lines of the breast and colon. In hematopoietic tissues, expression of PPARγ has been reported in bone marrow stromal cells, cluster of differentiation 34^+^ (CD34^+^) progenitor cells, normal monocyte/macrophage cells, lymphocytes and neutrophils which indicate that PPARγ plays an essential role in both erythropoiesis and adipogenesis (16, 17). Additionally PPARγ plays a pivotal role in regulation of hematopoietic stem cell (HSC) proliferation, osteoclastogenesis and monocyte differentiation (18).

PPARγ ligands have modulating effects on proliferation and differentiation of many cancer cell lines (8-10) therefore, some of the PPARγ ligands have been used in different and widespread clinical applications (19). Ligands such as troglitazone and pioglitazone suppress and postpone proliferation of primary erythroid progenitor cells (20). Troglitazone, a PPARγ agonist, is one of the drugs used as treatment for diabetes mellitus type 2 (21, 22). As with the other thiazolidinediones (TZDs) troglitazone activates PPAR and affects bone metabolism (23). Troglitazoneis a ligand for both PPARα and especially PPARγ. Baicalin herbal extract activates PPARγ and is considered as a treatment for many diseases due to its anticancer effects. Activation of PPARγ can modulate erythroid differentiation. This study has sought to assess the effects of PPARγ on differentiation, survival and proliferation of erythroid progenitor cells.

## Materials and Methods

### Purification and expansion of CD133^+^ umbilical cord blood cells

In this experimental study, five umbilical cord blood samples were obtained from normal infants of mothers who volunteered following informed consent. This study received the approval of the Ethical Committee at Tabriz University of Medical Sciences. For this purpose, fresh umbilical cords were used at the time of full-term delivery or maximally within four hours after delivery. Blood was diluted with hydroxyethyl starch (HES; HAES-sterile 6%, Sigma-Alderich, USA) and centrifuged at a final concentration of 2.5% in 12 mm diameter falcon tubes at 22℃ and 650 rpm. Then, the plasma layer that contained mononuclear cells was collected and after centrifugation, the cell sediment was suspended in phosphate-buffered saline (PBS; Sigma-Alderich, USA) solution, layered on Ficoll (Baharafshan, Iran, d: 1.077 g/ml) and centrifuged at 800 g for 35 minutes at 24˚C. The light density mononuclear cells were collected in Iscove’s modified Dulbecco medium (IMDM; Gibco BRL, NY, USA) incubated with CD133^+^ conjugated magnetic microbeads and stored at 4-8˚C for 30 minutes. After washing, the suspension was processed through the LS columns (Miltenyi Biotec, Germany) MACS magnetic separation column (Miltenyi Biotec, Bergisch Gladbach, Germany) to obtain purifi ed CD133^+^ cells by the positive selection method. The unlabelled cells were passed through the column. The column was then removed from the magnetic separator and the labeled cells were flushed out. Cells were turned into a suspension after centrifugation at 300 g for 10 minutes in 500 ml of liquid.

### HSC culture

CD133^+^ cells were cultivated in IMDM that contained 20% fetal bovine serum (FBS; stem cell technologies, Vancover, BC Canada) at 37˚C in 5% CO_2_ in a high-humidity incubator. The medium was contained recombinant human stem cell factor (rhSCF; 100 ng/mL, Genescript, USA), thrombopoietin (TPO; 100 ng/ml, Genescript, USA), Fms-like tyrosine kinase-3 ligand (FLT3L; 100 ng/ml, Genescript, USA), 100 U/ml of penicillin and 100 μg/ml of streptomycin (Gibco, NY, USA). The proliferated cells were seeded onto 96 well plates for erythroid differentiation in IMDM that contained 20 ng/ml stem cell factor (SCF; Stem cell technology, Vancover, BC Canada) and 4.5 U/ml erythropoietin (EPO; Genescript, USA). The medium was replaced every three days from the fourth day and fresh cytokines were added. In order to study the effects of the PPARγ agonists. Baicalin and troglitazone on erythroid differentiation, we tested for proliferation and differentiation of HSCs in the presence and absence of these materials. Baicalin and troglitazone (Sigma-Aldrich, USA) were dissolved in dimethyl sulfoxide (DMSO; Sigma-Aldrich, USA) and added to the HSC culture at the following concentrations of baicalin (1, 10, 20 and 50 μM) and troglitazone (0.1, 1, 3 and 10 μM).

### Assessment of cell differentiation by flow cytometry

In order to determine erythroid differentiation, CD71 expression [transferring receptor (TfR)] and glycoprotein A (GPA) on the surface of the erythroid differentiated cells were analyzed by flow cytometry. A total of 1×10^5^ cells were washed twice with ice-cold then incubated with anti-GPA monoclonal antibody conjugated with fluorescein isothiocyanate (FITC; Dako Cytomation, Denmark), and anti-CD71 monoclonal antibody conjugated with phycoerythrin (PE; Dako Cytomation, Denmark) at 37˚C for 30 minutes. Cells were subsequently analyzed with fluorescence-activated cell sorting (FACS) Calibur (Becton Dickinson, USA).

### Colony forming unit (CFU) assay

Ability of HSC cells to form colonies was studied by adding these cells to IMDM medium based on methyl cellulose (MC). In order to prepare MC medium, 26 g of autoclaved MC (Fisher, M-281, USA) was added to 500 ml of sterile distilled water (70-90˚C) and stirred for 10 minutes using a magnetic blade (Gemmy Industrial Corp, Taiwan). The gel formation process was started by the gradual addition of 500 mL of 2x IMDM medium to this medium, which resulted in gel formation at 4˚C within 6-24 hours. In order to study colony forming ability, 1×10^4^ cells were transferred to 100 μl of IMDM medium, 50 μM of baicalin and 3 μM of troglitazone. Cytokines of 4 U/mL recombinant human erythropoietin (rhEPO) and 20 ng/ml rhSCF were added to 1 ml of the prepared MC medium. Then, this medium was poured with a 16 G needle in to a cell culture plate. Cells were incubated at 37˚C in a humidifi ed incubator in 5% CO_2_ for 14 days. We added 200 ml of IMDM medium that contained the above mentioned drugs were every three days to prevent desiccation. Colony assay was compared with the control cells grown in the absence of agonists.

### Morphological study of erythroid differentiated cells

In order to study differentiation changes, we stained the cells with Wright-giemsa and observed them with a microscope.

### Assessment of the number of viable and nonviable cells by flow cytometry

Cells were cultured in presence of PPARγ agonists. The numbers of viable and nonviable cells were evaluated by harvesting a constant volume of cellular suspension from these cells and analyzing them by flow cytometry (FACSCalibur, Becton Dickinson, USA). Using the diagram of cellular characteristics in forward scatter (FS) and side scatter (SS), we calculated the numbers of viable and nonviable cells in the sample (24-26). Data were reported as mean ± standard deviation (SD) and analyzed by the student’s t test using statistical package for social science (SPSS) 22.0 software.

## Results

### Morphological study of erythroid differentiated cells

**Fig.1:**
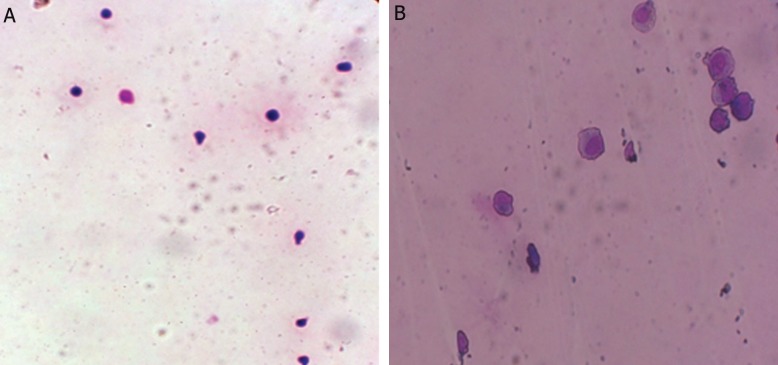
Wright-giemsa stained cells at day 7 of erythroid differentiation. A. Hematopoietic stem cells (HSCs) separated from the umbilical cord blood at day 0 (magnitude: ×10) and B. Erythroid progenitor cells at day 7 of differentiation (magnitude: ×40).

In order to study morphological changes of cells obtained from proliferation and differentiation of CD133^+^ into erythroid differentiated cells, Wright-giemsa staining was done and differentiation of progenitors into the erythroid lineage was investigated (Fig.1). The morphological studies confirmed erythroid differentiation of these cells. When CD133^+^ were cultured with baicalin and troglitazone, day-12 cells retained immature features, whereas CD133^+^ cultured without baicalin and troglitazone exhibited normal terminal erythroid maturation with nuclear condensation and enucleation.

### Effects of baicalin and troglitazone in TfR and GPA expression on erythroid differentiated cells

In order to determine the effects of PPARγ ligands on proliferation and differentiation of HSCs, CD133^+^ cells separated from the umbilical cord blood were incubated with different concentrations of baicalin (10, 20 and 50 μM) and troglitazone (0.1 and 1 μM) in IMDM that was contained 20% FBS. The percentage of erythroid cells was confi rmed by fl ow cytometric analysis for the erythroid markers GPA and TfR on day 7. The mean percentage of GPA expressing cells in the presence of 10 μM baicalin at day 7 was similar to that of the control group (84 ± 2%).

**Fig.2:**
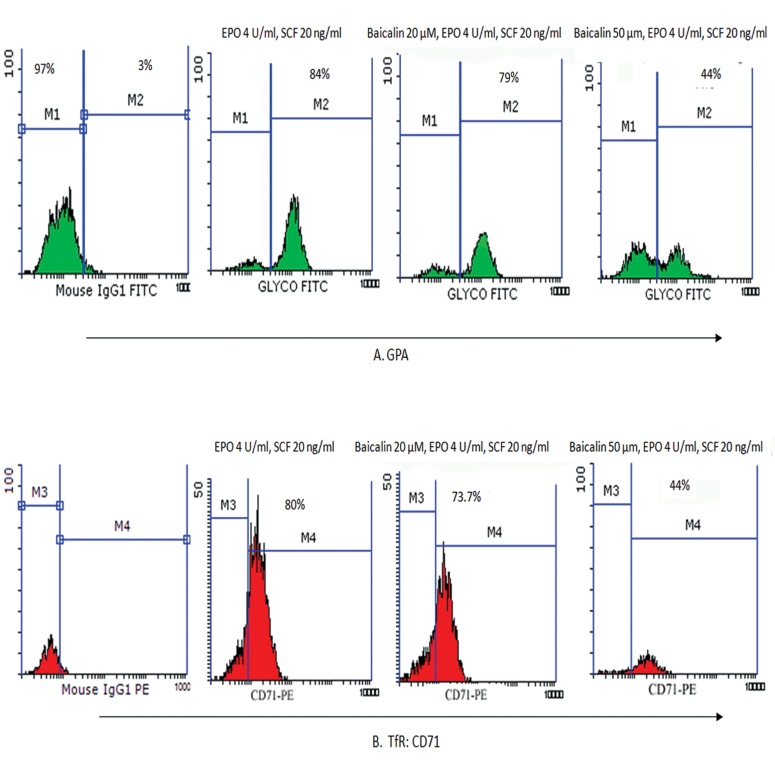
Expressions of GPA (A) and (B) CD71 surface markers evaluated by flow cytometry at day 7 of erythroid differentiation in IMDM-containing cytokines, SCF (20 ng/ml) and EPO (4.5 U/ml) in the presence of baicalin at concentrations of 20 and 50 μM and control isotypic antibody. GPA; Glycophorin A, CD; Cluster of differentiation, IMDM; Iscove’s modified dulbecco medium, SCF; Stem cell factor, EPO; Erythropoietin, M; Monocolonal, IgG; Imonoglubolin, FITC; Fluorescein isothiocyanate, TfR; Transferrin receptor and PE; Phycoerythrin.

**Fig.3:**
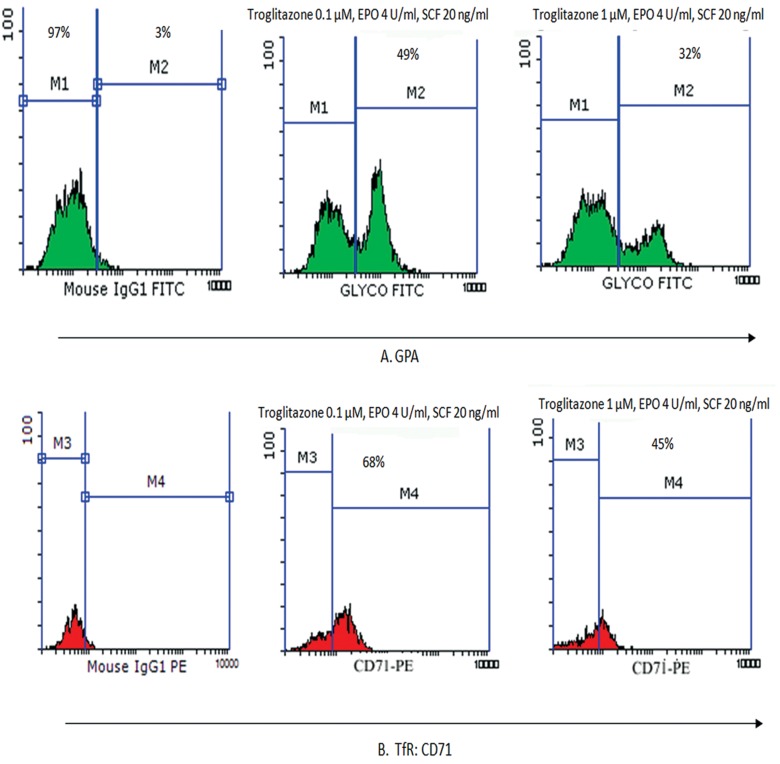
Expressions of GPA (A) and CD71 (B) surface markers evaluated by flow cytometry at day 7 of erythroid differentiation in IMDM that contained SCF (20 ng/ml) and EPO (4.5 U/ml) in the presence of troglitazone at concentrations of 0.1 and 10 μM and control isotypic antibody. GPA; Glycophorin A, CD; Cluster of differentiation, IMDM; Iscove’s modified dulbecco medium, SCF; Stem cell factor, EPO; Erythropoietin, M; Monocolonal, IgG; Imonoglubolin G, FITC; Fluorescein isothiocyanate, TfR; Transferrin receptor and PE; Phycoerythrin.

This value was 79.5 ± 4% for 20 μM of baicalin and 44 ± 3% for 50 μM of baicalin, which indicated a dose-dependent decrease in the percentage of GPA expressing cells (p<0.05, n=3, Fig.2). In addition, we observed a dose-dependent reduction in the percentage of TfR expressing cells. According to flow cytometry analyses, the mean percentages of TfR expressing cells were 80 ± 4% for 10 μM of baicalin, 73 ± 5% for 20 μM of baicalin, and 17 ± 3% for 50 μM of baicalin at day 7. The percentage of TfR expressing cells was similar to the control cells for GPA expression at 10 μM baicalin. A comparison of treatment with troglitazone on day 7, showed the mean percentage of cells that expressed the GPA erythroid marker were 49 ± 3% for 0.1 μM and 32 ± 5% at 1 μM. The mean percentages of CD71 marker were 68 ± 4% (0.1 μM) and 45 ± 2% for 1 μM (p<0.05, n=3, Fig.3). Therefore, a decrease in the percentage of cells that expressed surface receptors was observed in 50 μM of baicalin and 1 μM of troglitazone.

### Effect of baicalin on erythroid CFU compared with troglitazone

**Fig.4:**
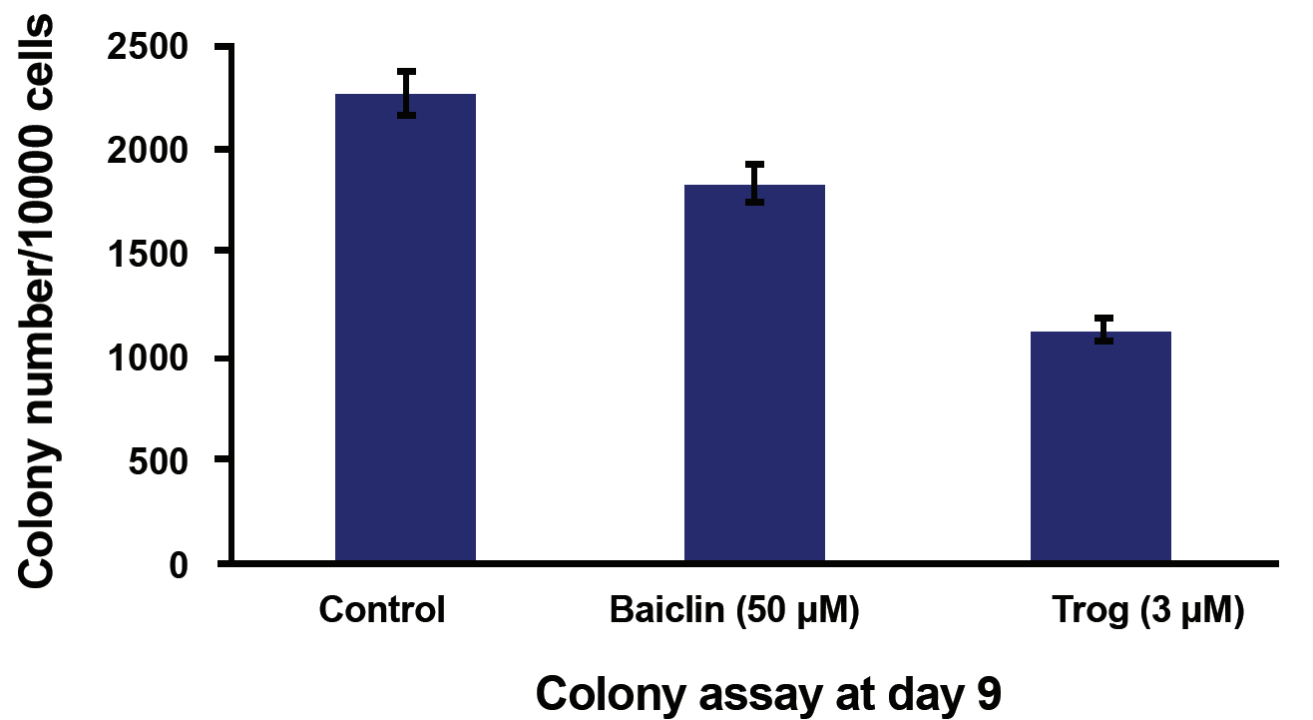
Effects of baicalin and (Trog) on CD133^+^ cell colony formation. The numbers of colonies that formed on the ninth day for 10000 primary cells was specified in the presence of baicalin (50 μM) and Trog (3 μM) by calibrating the culture plate and counting lens (magnitude: ×100). Trog; Troglitazone and CD; Cluster of differentiation.

**Fig.5:**
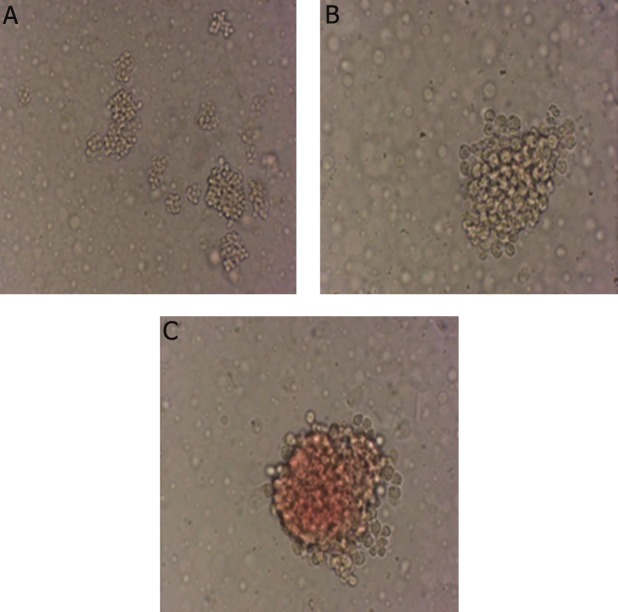
Colonies formed after 9 days in (MC) medium. A. Different colonies (magnitude: ×10), B. Granulocyte colony (magnitude: ×10) and C. Erythroid colony (magnitude: ×40). MC; Methyl cellulose.

We performed the colony-forming assay to further evaluate whether treatment with these reagents affected colony formation. Concentrations of baicalin (50 μM) and troglitazone (3 μM) that caused significant suppression of surface marker expressions were prepared and placed in an incubator at 5% CO_2_ along with the cytokine rhEPO (4 U/mL) and rhSCF (20 ng/mL). The colony assay test results were compared to the control group results. Colony numbers decreased after treatment with baicalin and troglitazone (p<0.05, n=3, Fig.4). Images of erythroid colonies that formed in semisolid medium after 9 days are shown in figure 5.

### Assessment of the number of viable and nonviable cells by flow cytometry

**Fig.6:**
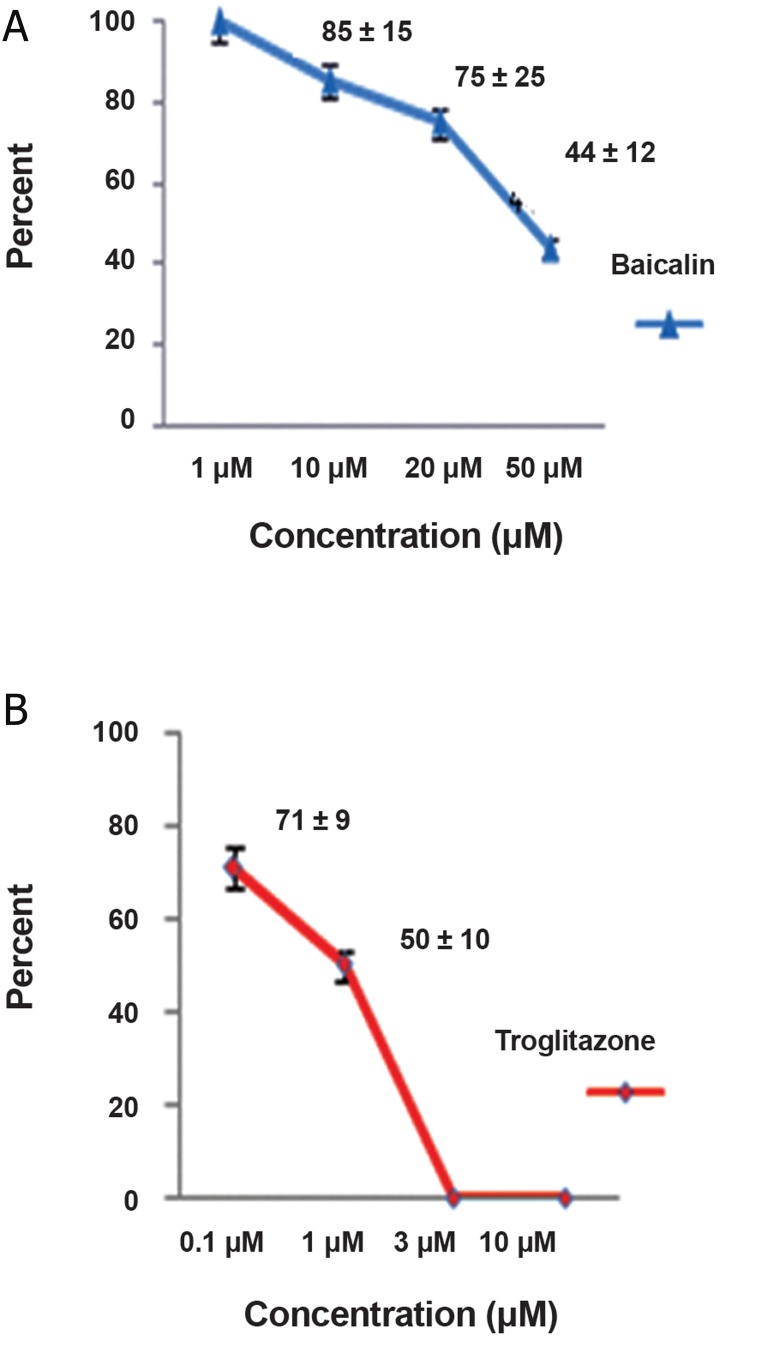
Percentage of viable cells treated with baicalin and troglitazone on day 7 by flow cytometry analysis: A. Percentage of viable cells against different concentrations of baicalin and B. Percentage of viable cells against different concentrations of troglitazone.

Incubation of CD133^+^ cells with PPARγ agonists (baicalin and troglitazone) reduced the number of viable cells. Evaluation was performed by harvesting a constant cell number from the cultures treated with the agonists at different concentrations. Cells were analyzed by flow cytometry. We used a histogram of cellular characteristics in FS and SS to calculate the numbers of viable and nonviable cells (24-26). Treatment CD133^+^ cells at various concentrations of baicalin showed the percentage of viable to nonviable cells on day 7 to be: 100% (1 μM), 85 ± 11% (10 μM), 75 ± 26% (20 μM) and 44 ± 12% (50 μM). There was a significant decrease at the 20 μM and lower concentrations (p<0.05, n=3). The percentages of viable cells in troglitazone were 71 ± 9% (0.1 μM) and 50 ± 10% (1 μM). There was a significant decrease at the 0.1 μM and less concentration (p<0.05, n=3, Fig.6). All cells were nonviable at concentrations of 3 and 10 μM and could not be evaluated by flow cytometry.

## Discussion

Numerous studies evaluated the effects of PPARγ ligands on different cells. PPARγ ligands have been shown to suppress cellular growth by induction of apoptosis in different human lymphoma and leukemic cell lines (19). In a study by Hirase et al. (27), it was shown that PPARγ synthetic ligands such as troglitazone and pioglitazone caused suppression of cellular proliferation as well decreased expression of GPA erythroid surface marker expressions in the K562 erythroleukemia cell line. In study by Nagasawa et al. (20), it was reported that thiazolidones postponed erythroid maturity and proliferation of erythroid colony-forming cells in HSCs separated from peripheral blood. It has been reported that hemoglobin and hematocrit decreased due to the use of thiazolidinones in diabetic patients (28, 29). Agonists such as troglitazone, pioglitazone and thiazolidones cause the induction of apoptosis in human colon cancer cells and breast cancer cells (30, 31). In the recent years, new synthetic derivatives of troglitazone with better anti-proliferative properties and lower toxic effects on various cancer cell lines have been used (31). In addition to troglitazone, natural ligands of PPARγ such as15dPGJ2 suppress cellular proliferation of ECFCs which is indicative of anti-proliferative properties mediated by PPARγ (20). Numerous studies have tested the effects of different agonists of PPARγ in cancer cell lines and erythropoiesis. In the present work, we studied the effect of baicalin on normal erythropoiesis. For the first time, the current study showed its suppressive effect on erythropoiesis.

The anticancer effects of baicalin and baicalein have been reported by several studies (6, 11, 24, 32-35). After cleavage of the glycoside moiety, baicalin is converted to baicalein *in vivo*. Baicalin causes G1 arrest and apoptosis of prostate cancer cells (11). In human hepatoma cells baicalin inhibits proliferation or causes apoptosis induction (10). Baicalin and baicalein have anti-potential angiogenesis effects (34). Anti-proliferative effects of baicalin on different cancer cell lines have been reported by different research groups. Also, the growth inhibiting effects of flavonoids such as baicalin were observed on human cancer cells and not on normal diploid cells (1). However, a study by Himeji et al. (8) showed that baicalin had a low toxic effect on normal cells in addition to its toxicity effects on cancer cells. Some studies reported that PPARγ ligands suppressed growth of cells in leukemic and lymphoma cell lines through induction of apoptosis (19, 36, 37). However, Nagasawa et al. (20) observed that PPARγ ligands suppressed proliferation of erythroid progenitor cells without decreasing survivability and increasing apoptosis.

The present study results revealed a considerable decrease in percentage of cells that expressed surface receptors in the presence of baicalin at a concentration of 50 μM and troglitazone at a concentration of 3 μM. The mean percentage of cells that expressed the GPA and TfR erythroid markers with baicalin was 84 ± 2% (10 μM), 79.5 ± 4 (20 μM) and 44 ± 3% (50 μM) for GPA at day 7 and 80 ± 4% (10 μM), 73 ± 5% (20 μM) and 17 ± 3% (50 μM) for TfR, which indicated that the number of cells which expressed these markers considerably decreased in a dose-dependent manner. This showed a delay in erythroid maturity of the progenitor cells. Prevalence of TfR expression was similar to that in the control sample and for GPA expression at the baicalin concentration of 10 μM which indicated that this concentration had no effect on differentiation of HSCs or their viability. In treatment with troglitazone as a PPARγ activator on day 7, the mean percentage of GPA erythroid marker expressing cells was 49 ± 3% (0.1 μM) and 32 ± 5% (1 μM) and the mean percentage of CD71 marker was 68 ± 4% (0.1 μM) and 45 ± 2% (1 μM). There were no viable cells at day 7 troglitazone concentrations of 3 and 10 μM. Studies showed that PPARγ ligands which included15-deoxy-Δ12, 14-prostaglandin J2, rosiglitazone and the novel triterpenoid 2-cyano-3,12-dioxooleana-1,9-dien-28-oic acid induced apoptosis in myeloid (U937 and HL-60) and lymphoid (Su-DHL, Sup-M2, Ramos, Raji, Hodgkin’s cell lines, and primary chronic lymphocytic leukemia) cells. A similar exposure to PPARγ ligands induced the differentiation of myeloid leukemic cells (19, 29).

This study found that the number of viable cells gradually decreased after the addition of baicalin while the number of nonviable cells in the group treated with the troglitazone agonist was higher. Therefore, sensitivity of cancer cells to baicalin extract was different. Sensitivity of normal cells such as HSC also differed during differentiation into the erythroid lineage (36). The proliferation of cells in the presence of troglitazone was suppressed to a greater extent compared with baicalin. Suppression of proliferation was enhanced when the concentration of these agonists increased (38). Asou et al. (39) showed that troglitazonein combination with a retinoid was a moderately potent inhibitor of clonogenic growth of acute myeloid leukemia cells. Moreover, recent results showed that troglitazone could inhibit cell proliferation and promote cell differentiation independent of PPARγ (40). In a comparison of the number of cells that expressed erythroid surface markers of GPA and TfR, it was determined that the number of cells that expressed these markers decreased in a dose-dependent manner when treated with baicalin and troglitazone compared to the control group. Results from the colony assay test indicated that the separated and proliferated CD133^+^ stem cells could quantitatively and qualitatively differentiate into different cellular lineages such as erythroids, which was confirmed by expressions of cell surface markers.

## Conclusion

Numerous reports have documented antibacterial, antiviral, anti-inflammatory and antitumor properties of baicalin in the field of innate immunity. Baicalin has been proposed as treatment for fever, allergy and inflammatory diseases as well as for treatment of neurodegenerative diseases and for its antiviral activity and anti-proliferative effects on different cell lines. However, the potential toxic effect of this extract on normal cells has not been well considered in these studies. Adverse effects are major obstacles associated with therapeutic agents used as treatments for human diseases. Findings of this research showed that baicalin had a considerable dose-dependent effect on erythroid differentiation of CD133^+^; therefore, these side effects as well as the dosage should be considered when prescribing this medication. This issue was noteworthy considering the plant origin of baicalin as well as different reports on its anticancer effects and its arbitrary publicuse.
